# Impact of the early phase of the COVID-19 pandemic on emergency department-to-intensive care unit admissions in Korea: an interrupted time-series analysis

**DOI:** 10.1186/s12873-024-00968-1

**Published:** 2024-04-01

**Authors:** Kyung-Shin Lee, Changwoo Han, Hye Sook Min, Jeehye Lee, Seok Hwa Youn, Younghwan Kim, Jae Young Moon, Young Seok Lee, Su Jin Kim, Ho Kyung Sung

**Affiliations:** 1 Public Health Research Institute, National Medical Center, 245 Eulgi-ro, Jung-gu, 04564 Seoul, Korea; 2 Department of Preventive Medicine, Chungnam National University College of Medicine, Daejeon, Korea; 3 Department of Preventive Medicine, Konkuk University College of Medicine, Chungju-si, Korea; 4 Department of Trauma Surgery, National Medical Center, Seoul, Korea; 5 Department of Pulmonary and Critical Care Medicine, Chungnam National University Sejong Hospital, Sejong, Korea; 6 Division of Pulmonology, Allergy and Critical Care Medicine, Department of Internal Medicine, Korea University Guro Hospital, Seoul, Korea; 7 Department of Emergency Medicine, Korea University Anam Hospital, Seoul, Korea; 8 Department of Population Health, NYU Grossman School of Medicine, New York, NY USA

**Keywords:** Intensive care unit, Emergency department, COVID-19, Hospital mortality, Interrupted time-series analysis

## Abstract

**Background:**

The coronavirus disease 2019 (COVID-19) pandemic resulted in significant disruptions to critical care systems globally. However, research on the impact of the COVID-19 pandemic on intensive care unit (ICU) admissions via the emergency department (ED) is limited. Therefore, this study evaluated the changes in the number of ED-to-ICU admissions and clinical outcomes in the periods before and during the pandemic.

**Methods:**

We identified all adult patients admitted to the ICU through level 1 or 2 EDs in Korea between February 2018 and January 2021. February 2020 was considered the onset point of the COVID-19 pandemic. The monthly changes in the number of ED-to-ICU admissions and the in-hospital mortality rates before and during the COVID-19 pandemic were evaluated using interrupted time-series analysis.

**Results:**

Among the 555,793 adult ED-to-ICU admissions, the number of ED-to-ICU admissions during the pandemic decreased compared to that before the pandemic (step change, 0.916; 95% confidence interval [CI] 0.869–0.966], although the trend did not attain statistical significance (slope change, 0.997; 95% CI 0.991–1.003). The proportion of patients who arrived by emergency medical services, those transferred from other hospitals, and those with injuries declined significantly among the number of ED-to-ICU admissions during the pandemic. The proportion of in-hospital deaths significantly increased during the pandemic (step change, 1.054; 95% CI 1.003–1.108); however, the trend did not attain statistical significance (slope change, 1.001; 95% CI 0.996–1.007). Mortality rates in patients with an ED length of stay of ≥ 6 h until admission to the ICU rose abruptly following the onset of the pandemic (step change, 1.169; 95% CI 1.021–1.339).

**Conclusions:**

The COVID-19 pandemic significantly affected ED-to-ICU admission and in-hospital mortality rates in Korea. This study’s findings have important implications for healthcare providers and policymakers planning the management of future outbreaks of infectious diseases. Strategies are needed to address the challenges posed by pandemics and improve the outcomes in critically ill patients.

**Supplementary Information:**

The online version contains supplementary material available at 10.1186/s12873-024-00968-1.

## Background

The coronavirus disease 2019 (COVID-19) pandemic has substantially disrupted critical care systems worldwide, including a marked decline in emergency department (ED) presentations in regions with widespread transmission of severe acute respiratory syndrome coronavirus 2 (SARS-CoV-2), such as the United States (US), United Kingdom, and Europe [1–7]. A study conducted in the US revealed a 42% reduction in the number of ED presentations due to all causes during the first wave of the pandemic compared to the corresponding period in 2019 [6]. An Italian study indicated that the decrease in the number of ED visits persisted during the second wave of the pandemic [7]. In Korea, a sudden surge in the number of patients with COVID-19 was noted from February to March 2020 [8], which corresponded to a decrease in the number of patients visiting EDs [9].

The decrease in the number of ED presentations during the COVID-19 pandemic raises concerns that patients with acute life-threatening conditions, such as acute myocardial infarction, stroke, and out-of-hospital cardiac arrest may not have received prompt medical attention [3]. In recent decades, the ED has emerged as a crucial point of entry for critically ill patients seeking intensive care unit (ICU) admission [10]. US-based studies have indicated that the number of ICU admissions from EDs has risen at a higher rate than that of the number of overall ED visits [11, 12]. In Korea, before the outbreak of the pandemic, over 200,000 critically ill patients were admitted to ICUs through EDs annually, accounting for 2.4% of all ED visits [13].

Although EDs mark a crucial route for unplanned ICU admissions, research on the impact of the COVID-19 pandemic on such admissions (from the ED to the ICU) is limited. This research is essential because critically ill patients in the ED can receive evidence-based interventions to ensure optimal outcomes [10]. In addition, the impact of the pandemic on critical care outcomes such as mortality is unclear [14]. The outcomes of patients requiring ICU management during a pandemic are affected by several factors, including patient characteristics, the organizational capacity of the critical care system, and the public health response to the pandemic [15, 16]. Therefore, we aimed to evaluate the changes in ED-to-ICU admissions at the national level during the early phase of the COVID-19 pandemic. Moreover, it aimed to investigate the variability of these changes across demographic and clinical characteristics and evaluate changes in critical care outcomes.

## Methods

### Data source and setting

We conducted a retrospective time-series analysis of data obtained from the National Emergency Department Information System (NEDIS) between February 2018 and January 2021. The NEDIS is an ED-based database established with the objective of evaluating the emergency care system in Korea, in accordance with Article 15 of the Emergency Medical Service Act. It collects data from all patients presenting to participating EDs nationwide, including demographic information, mode of arrival, date and time of ED arrival and departure, triage scores, vital signs upon ED arrival, diagnostic codes, ED disposition, and clinical outcomes. Data transmitted from the EDs to the NEDIS were processed, and all patient-related information were anonymized and checked for data integrity. In Korea, EDs are classified into three levels based on their capacity and capability: level 1, regional emergency centers; level 2, local emergency centers; and, level 3, local emergency facilities [17]. Due to limited resources, level 3 EDs are less capable of providing critical care than are level 1 or level 2 EDs, and patient assessment information, such as triage scores and vital signs, provided to the NEDIS is typically missing or incomplete. Throughout the study period, the participation rate of EDs contributing to the NEDIS data remained consistently high: 99.5% in 2018, 99.8% in 2019, and 100% in 2020 and 2021 (Supplementary Table 1 [see Additional file 1]). The detailed design and variables of the NEDIS are described elsewhere [9, 13, 18, 19].

### Study period and population

From the NEDIS database, we identified ED-to-ICU admissions between February 1, 2018, and January 31, 2021, based on the date of presentation to the ED. The first case of COVID-19 was reported in Korea on January 20, 2020 [8]. However, the number of ED presentations decreased sharply after the first COVID-19 wave in February 2020 (Supplementary Fig. 1 [see Additional file 1]) [18]. Therefore, the period from February 2020 to January 2021 was designated as the COVID-19 pandemic. During the 12-month period, 78,197 COVID-19 cases were reported in Korea. The duration from February 2018 to January 2020 was designated as the pre-pandemic period. The study period was set based on a prior study that recommends inclusion of at least 12 data points before and after the event to allow for significant adjustment for seasonality when using monthly time-series data [20].

Patients with incomplete information on age or sex, those < 18 years old, or those with missing information on ED presentation time and date were excluded from the study. Additionally, patients admitted to the ICU through level 3 EDs were excluded because these EDs reported different data collection methods, patient characteristics, and outcomes compared to other ED levels [13]. Since this study was conducted with patients who were admitted to the ICU through the ED alive, cases of in-ED mortality or cardiopulmonary arrest on arrival were not included.

### Study outcomes and measurements

The primary outcome was the number of ICU admissions to the ED before and during the pandemic. The secondary outcome was in-hospital mortality.

Demographic and clinical data were collected from the NEDIS database, including age, sex, insurance type, injury upon ED presentation, emergency medical services (EMS) presentation, transferred-in, initial triage score, National Early Warning Score (NEWS) on presentation, ED length of stay (LOS), diagnostic codes, Charlson Comorbidity Index (CCI) score, and discharge status. The initial triage was performed using the Korean Triage and Acuity Scale (KTAS), which ranks patients according to clinical acuity on a scale of 1 to 5, where 1 indicates the need for immediate resuscitation and 5 indicates non-urgent care [21]. The NEWS was calculated using six physiological parameters, viz. respiratory rate, oxygen saturation, blood pressure, pulse rate, level of consciousness, and body temperature, with two additional points for patients requiring supplemental oxygen [22]. The NEWS values were categorized into three groups: 0–4, 5–6, and ≥ 7 [23]. ED LOS was defined as the time interval between a patient’s arrival at the ED and their departure. Prolonged ED LOS was defined as an ED LOS of ≥ 6 h and has been associated with increased mortality risk and a negative effect on the quality of care for critically ill patients in the ED [13, 24]. The diagnostic codes used during hospitalization were based on the International Classification of Diseases, Tenth Revision (ICD-10). The CCI was calculated using previously established methods [25, 26] based on the diagnostic codes used during hospitalization.

### Statistical analysis

Descriptive analyses were employed to compare the patient characteristics before and during the pandemic. Categorical variables are presented as frequencies and proportions and compared between patient groups using the Pearson chi-square test. Continuous variables were presented as the median and interquartile range (IQR) and analyzed using the Wilcoxon rank–sum test. We also calculated the numbers of ED-to-ICU admissions and in-hospital deaths for the ten most common primary diagnoses before and during the pandemic.

To investigate the potential effect of the COVID-19 pandemic on each outcome, we conducted an interrupted time-series analysis using a quasi-Poisson regression model and estimated the relative risk (RR) of abrupt steps and slope changes in outcomes over both periods (before and during the pandemic) [27, 28]. The data was aggregated monthly to reduce data fluctuations. To account for seasonal variations, we included the harmonic functions of the calendar month variables in the model [29]. The model is as follows:

$${Y_t} = {\beta _0} + {\rm{ }}{\beta _1}{T_t} + {\beta _2}{X_t} + {\beta _3}({T_t} - {T_0}) \cdot {X_t}$$

where Y_t_ represents the number of ICU admissions as the outcome, β_0_ represents the baseline level, β_1_ represents the time since the start of the study (in months), β_2_ is the level change following the intervention, which is an indicator variable for the pandemic (X_t_ = 0: before the pandemic; X_t_ = 1: during the pandemic), and β_3_ indicates the slope change following the intervention (with T_0_ as the time elapsed from the beginning of the COVID-19 pandemic). Harmonic terms specifying the number of sine and cosine pairs were used to adjust for seasonality [30]. β_2_ and β_3_ estimated the RR of abrupt steps and slope changes in the number of ICU admissions during the first year of the COVID-19 pandemic, respectively. Here, a step change denotes an abrupt and sustained alteration in time series data, while a slope change refers to a shift in the trend of the time series data [28]. Furthermore, we conducted stratified analysis to explore whether there are differences in the number of ICU admissions and the proportion of in-hospital mortality based on patient characteristics.

All analyses were performed using SAS 9.4 (SAS Inc., Cary, NC, USA) and R version 4.1.3 (R Foundation for Statistical Computing, Vienna, Austria). All tests were two-tailed, and *P* values < 0.05 were considered statistically significant.

## Results

A total of 555,793 adult ED-to-ICU admissions were identified from the NEDIS database between February 2018 and January 2021. Of these, 374,560 (67.4%) patients were admitted within 24 months before the COVID-19 pandemic, and 181,233 (32.6%) patients were admitted within 12 months of the pandemic. The number of presentations in level 1 or 2 EDs, the number of ED-to-ICU admissions, and the ED-to-ICU admission rate per month during the study period are shown in Supplementary Table 2 [see Additional file 1].

The characteristics of the patients who underwent ED-to-ICU admissions before and during the COVID-19 pandemic are presented in Table 1. Sex did not differ significantly between the groups. The proportion of patients transferred from other hospitals decreased during the COVID-19 pandemic (36.6% before vs. 30.6% during the pandemic; *P* value < 0.001). The NEWS values of the study population at ED presentation were also lower during than those before the pandemic. The median NEWS was 4 (IQR 1–6) before the pandemic and 3 (IQR 1–6) during the pandemic. The NEWS was ≥ 7 in 19.1% of patients before the pandemic and 17.6% during the pandemic. Compared to the pre-pandemic period, the median ED LOS during the COVID-19 pandemic was significantly longer (3.7; IQR 2.1–6.8 h before vs. 4.3; IQR 2.4–8.6 h during, *P* value < 0.001). The monthly median ED LOS and percentage of prolonged ED LOS for the study patients are presented in Supplementary Fig. 2 [see Additional file 1]. The face validity of the ten most common primary diagnoses did not differ before and during the pandemic period (Supplementary Table 3 [see Additional file 1]). Acute myocardial infarction was the most common primary diagnosis in adult patients who underwent ED-to-ICU admissions, both before and during the pandemic (9.5% before vs. 9.6% during the pandemic, *P* value = 0.573). The frequency of cerebral infarction, intracerebral hemorrhage, sepsis, and acute renal failure increased, while that of intracranial injury, pneumonia, and cardiac arrest decreased, among all ED-to-ICU admissions during the pandemic. The in-hospital mortality rate for ED-to-ICU admissions was 14.3% in the pre-pandemic period, which significantly rose to 14.7% during the pandemic period (*P* value < 0.001). The top ten primary diagnoses with the highest number of deaths among patients with ED-to-ICU admission before and during the COVID-19 pandemic are presented in Supplementary Table 4 [see Additional file 1].

**Table 1:** Characteristics of patients admitted to the ICU through the ED before and during the pandemic

	Before the COVID-19 pandemic (*n* = 374,560)	During the COVID-19 pandemic (*n* = 181,233)	*P* value
Age, year			
Median (IQR)	69 (56–79)	69 (56–79)	< 0.001
Mean (SD)	66.4 (16.0)	66.6 (16.1)	< 0.001
18–44	37,066 (9.9)	17,754 (9.8)	
45–64	121,503 (32.4)	57,824 (31.9)	
65–79	126,595 (33.8)	60,463 (33.4)	
80 or older	89,396 (23.9)	45,192 (25.0)	
Female	149,608 (40.0)	72,106 (39.8)	0.266
Insurance type			
NHI	323,876 (86.5)	156,856 (86.6)	< 0.001
Medical Aid	42,808 (11.4)	21,281 (11.7)	
Uninsured or other	7,876 (2.1)	3,096 (1.7)	
EMS presentation	272,883 (72.9)	132,610 (73.2)	0.013
Transferred-in	136,934 (36.6)	55,456 (30.6)	< 0.001
Injury-related presentation	62,223 (16.6)	29,328 (16.2)	< 0.001
KTAS score			
1	39,619 (10.6)	18,336 (10.1)	< 0.001
2	135,793 (36.3)	62,290 (34.4)	
3	169,828 (45.3)	87,641 (48.4)	
4	26,481 (7.1)	11,715 (6.5)	
5	2,796 (0.8)	1,246 (0.7)	
Unidentified	43 (0.0)	5 (0.0)	
NEWS			
Median (IQR)	4 (1–6)	3 (1–6)	< 0.001
Mean (SD)	4.1 (3.4)	4.0 (3.3)	< 0.001
0–4	186,212 (49.7)	91,694 (50.6)	< 0.001
5–6	50,602 (13.5)	23,553 (13.0)	
7 or more	71,534 (19.1)	31,922 (17.6)	
Unidentified	66,212 (17.7)	34,064 (18.8)	
ED Length of stay, h			
Median (IQR)	3.7 (2.1–6.8)	4.3 (2.4–8.6)	< 0.001
Mean (SD)	6.8 (8.5)	7.6 (8.7)	< 0.001
6 or more	108,830 (29.1)	66,072 (36.5)	< 0.001
CCI score			
Median (IQR)	1 (0–1)	1 (0–1)	< 0.001
Mean (SD)	1.0 (1.2)	1.1 (1.2)	< 0.001
0	151,839 (40.5)	71,569 (39.5)	< 0.001
1	135,323 (36.1)	64,891 (35.8)	
2	43,481 (11.6)	21,960 (12.1)	
3 or more	43,917 (11.7)	22,813 (12.6)	
Level of ED			
I	174,101 (46.5)	84,320 (46.5)	0.756
II	200,459 (53.5)	96,913 (53.5)	
In-hospital mortality	53,389 (14.3)	26,658 (14.7)	< 0.001

Data are presented as numbers (%), unless otherwise indicated

ICU, intensive care unit; ED, emergency department; COVID-19, coronavirus disease 2019; IQR, interquartile range; SD, standard deviation; NHI, national health insurance; EMS, emergency medical service; KTAS, Korean triage and acuity scale; NEWS, National Early Warning Score; CCI, Charlson Comorbidity Index

**Fig. 1:**
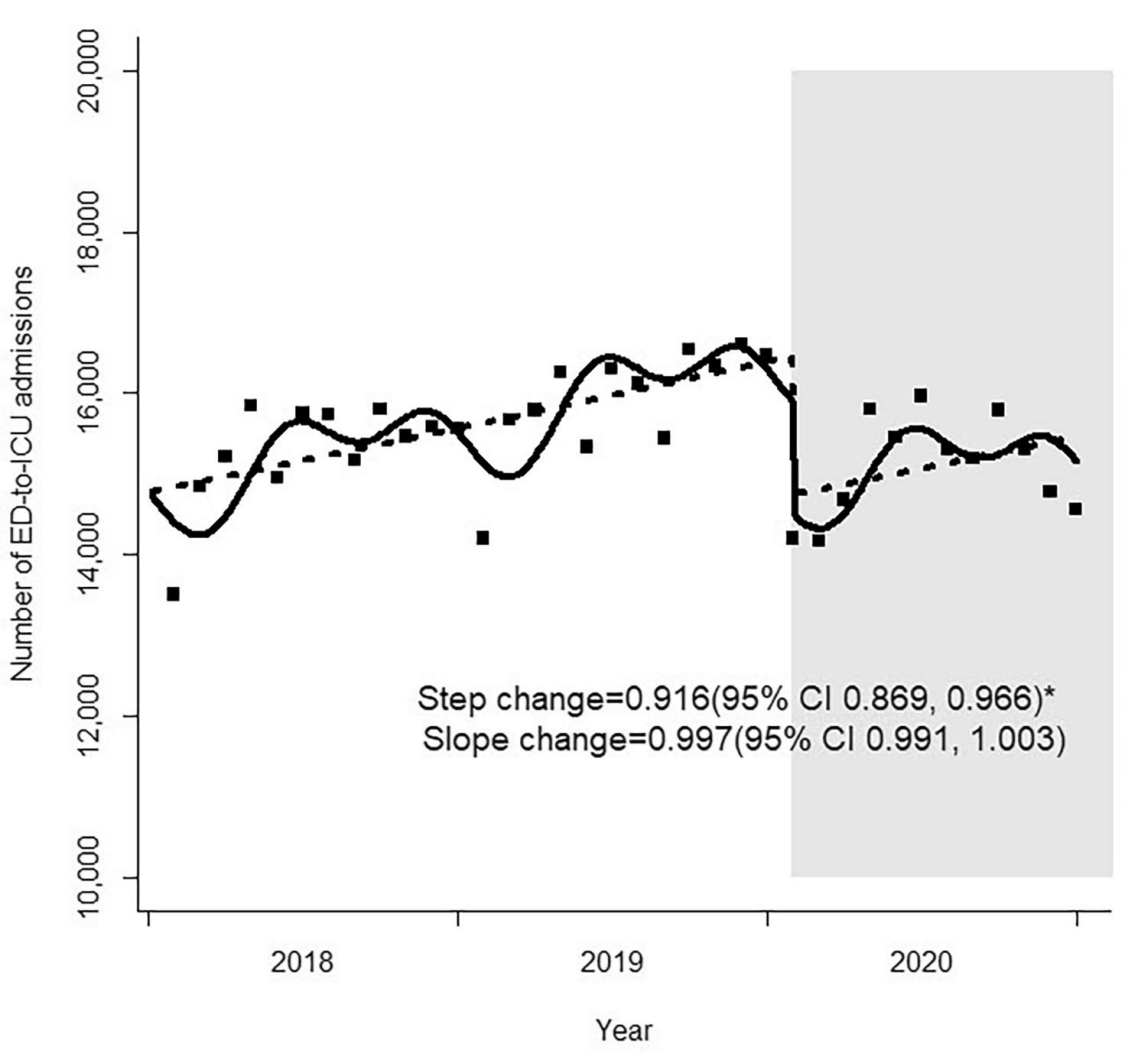
Monthly number of ED-to-ICU admissions before and during the COVID-19 pandemic. ED, emergency department; ICU, intensive care unit; COVID-19, coronavirus disease 2019; CI, confidence interval

**Fig. 2:**
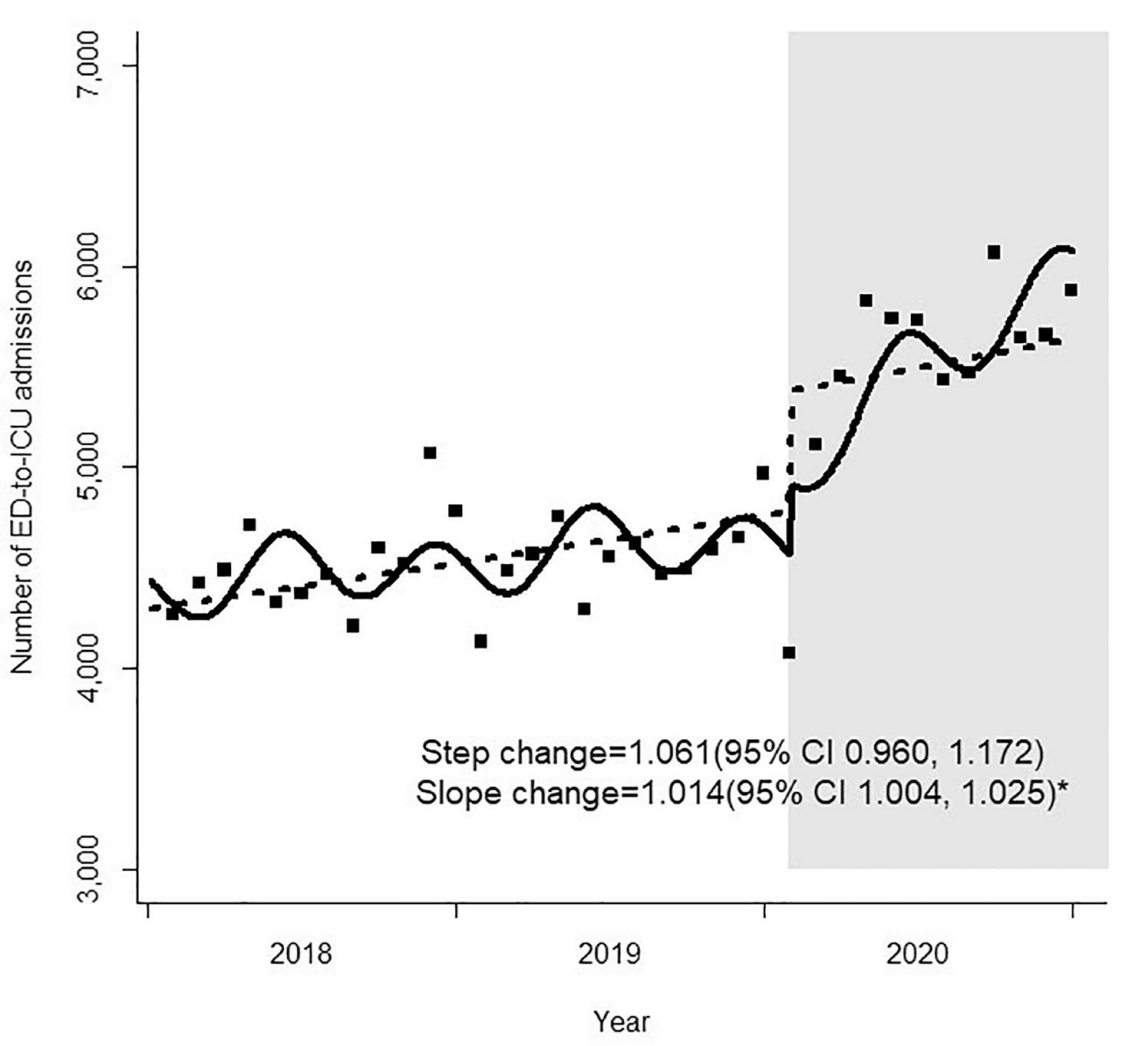
Monthly number of prolonged ED LOS among ED-to-ICU admissions before and during the COVID-19 pandemic. ED, emergency department; LOS, length of stay; ICU, intensive care unit; COVID-19, coronavirus disease 2019; CI, confidence interval

The risk estimates for changes in monthly ED-to-ICU admissions before and during the COVID-19 pandemic are presented in Table 2. The number of ED-to-ICU admissions plummeted abruptly during the pandemic compared to the pre-pandemic period (step change, 0.916; 95% confidence interval [CI] 0.869–0.966), although the trend did not attain statistical significance (slope change, 0.997; 95% CI 0.991–1.003) (Fig. 1). Stratification analysis by age group and sex yielded similar results. ED-to-ICU admissions of patients who arrived by EMS, those transferred from other hospitals, and patients with injuries showed decreased significantly during the pandemic. The decrease in the step change was 0.926 for patients arriving by EMS (95% CI 0.877–0.978), 0.846 for patients transferred from other hospitals (95% CI 0.789–0.908), and 0.920 for patients with injuries (95% CI 0.865–0.977). However, these trends did not change significantly over time. Significant step reductions were observed in patients with KTAS scores 1 (step change, 0.918; 95% CI 0.858–0.983) and 2 (step change, 0.862; 95% CI 0.813–0.915), which are associated with relatively higher acuity. However, this decrease was not observed in low-acuity patients. Analysis using the physiological index showed abrupt step reductions in NEWS 0–4 (step change, 0.883; 95% CI 0.838–0.930) and 5–6 (step change, 0.881; 95% CI 0.828–0.937). No significant step changes were observed in patients with NEWS ≥ 7, although there was a slope change (slope change, 0.989; 95% CI 0.980–0.999). There was no significant step change in patients with an ED LOS of ≥ 6 h until admission to the ICU. However, there was an increasing trend in ICU admission in these patients (slope change, 1.014; 95% CI 1.004–1.025) (Fig. 2). Analysis by ED level revealed that both level 1 and 2 EDs showed a drop in ED-to-ICU admissions during the pandemic.

**Table 2:** Interrupted time-series analysis of the monthly number of ED-to-ICU admissions before and during COVID-19 pandemic

	Step change	95% CI	Slope change	95% CI
Overall study population	0.916	0.869–0.966	0.997	0.991–1.003
Age, year				
18–44	0.920	0.851–0.994	0.999	0.991–1.008
45–64	0.933	0.885–0.984	0.994	0.988–1.000
65–79	0.912	0.855–0.972	1.001	0.994–1.008
80 or older	0.899	0.846–0.955	0.995	0.988–1.002
Sex				
Male	0.928	0.879–0.981	0.997	0.991–1.003
Female	0.898	0.850–0.950	0.997	0.991–1.004
Insurance type				
NHI	0.912	0.865–0.963	0.998	0.992–1.004
Medical Aid	0.934	0.874–0.998	0.992	0.985–0.999
EMS presentation	0.926	0.877–0.978	0.996	0.991–1.002
Transferred from other hospitals	0.846	0.789–0.908	0.991	0.983–0.999
Injury-related presentation	0.920	0.865–0.977	0.995	0.988–1.002
KTAS score				
1	0.918	0.858–0.983	0.995	0.987–1.002
2	0.862	0.813–0.915	1.002	0.996–1.009
3	0.950	0.898–1.005	0.996	0.989–1.002
4	0.969	0.913–1.027	0.982	0.976–0.989
5	0.930	0.769–1.125	0.992	0.971–1.014
NEWS				
0–4	0.883	0.838–0.930	0.999	0.994–1.005
5–6	0.881	0.828–0.937	0.993	0.986–1.000
7 or more	0.935	0.860–1.017	0.989	0.980–0.999
Unidentified	1.024	0.951–1.102	1.002	0.994–1.010
Prolonged ED LOS	1.061	0.960–1.172	1.014	1.004–1.025
CCI score				
0	0.900	0.841–0.962	0.996	0.989–1.003
1	0.919	0.865–0.976	0.998	0.991–1.005
2	0.931	0.879–0.985	0.999	0.993–1.005
3 or more	0.951	0.892–1.014	0.996	0.989–1.003
Level of ED				
I	0.937	0.880–0.997	0.995	0.988–1.002
2	0.899	0.852–0.949	0.999	0.993–1.005

ICU, intensive care unit; ED, emergency department; CI, confidence interval; COVID-19, coronavirus disease; NHI, national health insurance; EMS, emergency medical service; KTAS, Korean triage and acuity scale; NEWS, National Early Warning Score; LOS, length of stay; CCI, Charlson Comorbidity Index

**Fig. 3:**
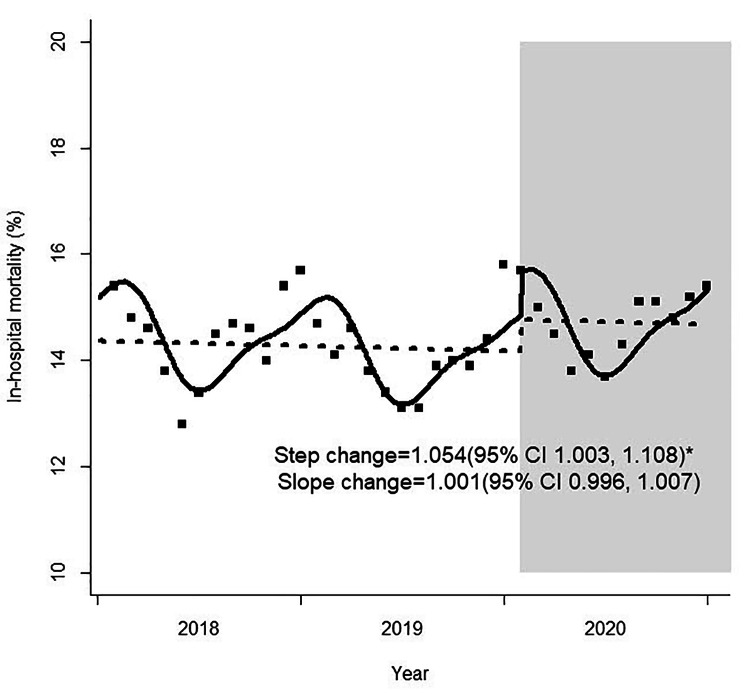
Monthly in-hospital mortality rates among ED-to-ICU admissions before and during the COVID-19 pandemic. ED, emergency department; ICU, intensive care unit; COVID-19, coronavirus disease 2019; CI, confidence interval

The risk estimates for changes in the monthly proportion of in-hospital mortality among ED-to-ICU admissions before and during the COVID-19 pandemic are presented in Table 3. There was a significant increase in the frequency of in-hospital deaths during the pandemic compared to the pre-pandemic period (step change, 1.054; 95% CI 1.003–1.108), but the trend did not change significantly (slope change, 1.001; 95% CI 0.996–1.007) (Fig. 3). The mortality rate in patients with an ED LOS of ≥ 6 h until admission to the ICU showed an abrupt increase after the pandemic (step change, 1.169; 95% CI 1.021–1.339). The mortality rate did not change significantly before or during the pandemic when patients were stratified by ED level. Multivariate analysis for patient characteristics showed that age, sex, insurance type, EMS presentation, injury-related presentation, KTAS score, NEWS, prolonged ED LOS, and CCI score were independently associated with in-hospital mortality both before and during the COVID-19 pandemic (Supplementary Table 5 [see Additional file 1]).

**Table 3:** Interrupted time-series analysis of the monthly in-hospital mortality rate before and during the COVID-19 pandemic

	Step change	95% CI	Slope change	95% CI
Overall study population	1.054	1.003–1.108	1.001	0.996–1.007
Age, year				
18–44	0.911	0.761–1.091	0.997	0.977–1.017
45–64	1.047	0.944–1.162	0.993	0.982–1.005
65–79	0.926	0.820–1.046	1.004	0.991–1.017
80 or older	0.952	0.884–1.025	0.997	0.989–1.005
Male	0.955	0.864–1.057	1.001	0.990–1.012
Female	0.973	0.898–1.055	0.995	0.987–1.004
Insurance type				
NHI	0.949	0.869–1.037	0.999	0.990–1.009
Medical Aid	1.019	0.913–1.138	0.993	0.981–1.005
EMS presentation	0.970	0.894–1.053	0.998	0.989–1.007
Transferred-in	0.866	0.768–0.977	0.988	0.975–1.002
Injury-related presentation	0.956	0.815–1.121	1.006	0.989–1.024
KTAS score				
1	0.952	0.864–1.049	0.998	0.987–1.008
2	0.914	0.832–1.005	1.001	0.991–1.011
3	1.019	0.914–1.135	0.996	0.985–1.008
4	1.039	0.842–1.282	0.996	0.974–1.019
5	0.821	0.452–1.492	1.042	0.977–1.110
NEWS				
0–4	0.900	0.813–0.996	1.003	0.992–1.014
5–6	0.936	0.835–1.049	0.998	0.986–1.011
7 or more	0.980	0.872–1.101	0.992	0.979–1.005
Unidentified	1.031	0.925–1.15	1.003	0.991–1.014
Prolonged ED LOS	1.169	1.021–1.339	1.012	0.998–1.026
CCI score				
0	0.955	0.843–1.081	0.995	0.981–1.008
1	0.948	0.862–1.043	1.004	0.994–1.015
2	1.017	0.902–1.147	0.997	0.984–1.010
3 or more	0.981	0.880–1.094	0.998	0.987–1.010
Level of ED				
I	0.962	0.874–1.058	0.997	0.986–1.007
2	0.963	0.874–1.062	1.000	0.990–1.011

CI, confidence interval; COVID-19, coronavirus disease; NHI, national health insurance; EMS, emergency medical service; KTAS, Korean triage and acuity scale; NEWS, National Early Warning Score; ED, emergency department; LOS, length of stay; CCI, Charlson Comorbidity Index

## Discussion

We investigated the changes in ED-to-ICU admission and mortality rates in Korea before and during the COVID-19 pandemic. The number of ED-to-ICU admissions decreased significantly during the pandemic. This decrease was observed in all age groups and both sexes but was most pronounced in patients who arrived by EMS, were transferred from other hospitals, had injuries, and had high-acuity conditions. The proportion of in-hospital deaths among ED-to-ICU admissions increased during the pandemic, especially in patients with an ED LOS ≥ 6 h before admission to the ICU. During the early phases of the pandemic, changes in ED-to-ICU admissions and mortality rates were reported in several countries; the magnitude and direction of these changes varied regionally. For instance, ED-to-ICU admissions increased in the US [31] but decreased in Canada [32]. ICU mortality rates during the pandemic also manifested regional differences [14]. These variations are attributed to a combination of factors such as the extent of COVID-19 spread, burden on the healthcare system, changes in patient healthcare-seeking behavior, regional healthcare infrastructure, and policies [14].

Several factors may have contributed to the decline in ED-to-ICU admissions during the first year of the pandemic. This decline can be partly explained by a decrease in demand. For example, patients with serious illnesses may have avoided visiting the ED because of the fear of contracting COVID-19. Korea experienced a large-scale nosocomial infection outbreak during the Middle East respiratory syndrome outbreak in 2015 [33], and the spread of COVID-19 within hospitals was also reported [34]. Fear of infection and the call for patients with COVID-19 to avoid visiting the ED may have impeded access to urgent health needs [35]. Surveys conducted in Korea in 2020 reported public avoidance of medical care and delayed access [36, 37]. Another possibility is that the implementation of non-pharmaceutical interventions (NPIs) such as working from home, travel restrictions, and bans on social gathering could have led to a decrease in outdoor activities and public transportation use [38], which could explain the decrease in ICU admissions for traumatic injury. A Korean study reported a decrease in the incidence and severity of injuries after the introduction of NPIs [39]. However, the reduction in demand is unlikely to account for the entire magnitude of decline. Public health measures to divert resources to patients with COVID-19 may have contributed to the decrease in ED-to-ICU admissions. For example, the Korean government implemented measures to accommodate patients with COVID-19 while also preventing the spread of the virus in hospitals. These measures included mandatory polymerase chain reaction tests for SARS-CoV-2 in patients presenting to the ED with fever or respiratory symptoms [40] and the designation of a portion of ICU beds exclusively for critically ill patients with COVID-19 [41, 42]. However, these measures may have hindered patients with non-COVID-19 critical conditions from accessing ICU care. Additional interviews and new COVID-19 testing protocols may have prolonged ED LOS, limiting ED capacity [40, 41]. In fact, some ICU beds (up to 4% of all ICU beds) were reserved for patients with COVID-19 and were unavailable to non-COVID-19 patients even if they remained unoccupied, which may have increased the threshold for ICU admission [41]. We found that the ED LOS of patients admitted to the ICU during the pandemic was longer than that of patients admitted to the ICU in the pre-pandemic period. This finding is consistent with that of other studies indicating output dysfunction of critical care in the ED [43, 44]. EMS capacity also decreased when medical resources were reallocated. Some ambulances and paramedics were dedicated to transporting patients with COVID-19 [27, 45], which may have contributed to the reduction in the numbers of patients transferred from other hospitals and those who arrived by EMS.

Despite the decrease in the number of ICU admissions from the ED during the pandemic, the in-hospital mortality rate increased during this period. A pandemic can alter the characteristics of patients admitted to the ICU. A US-based study reported that the proportion of patients admitted to the ICU for respiratory insufficiency and sepsis increased during the pandemic surge period compared to that before the pandemic period, whereas the proportion of patients diagnosed with myocardial infarction and stroke decreased [46]. In contrast, a study in Japan reported that the etiologies of patients in the ICU were similar before and during the COVID-19 pandemic [47]. In our study, the primary diagnoses before and during the pandemic were similar, and the degree of physiological deterioration at the time of ED presentation, as measured by the NEWS, was reduced. One possible explanation is that prolonged ED LOS may have contributed to the rise in mortality observed during the pandemic. Prolonged ED LOS is potentially dangerous for critically ill patients, as EDs may not have the necessary equipment or staff to provide the complex and continuous care required by critically ill patients [10]. A study conducted in Korea before the pandemic reported that ED LOS of ≥ 6 h in patients admitted to the ICU from the ED was associated with a higher risk of mortality [13], while other studies have reported a dose-response relationship between ED LOS and the risk of mortality [48–50]. The dose-response relationship observed in prior studies could also explain the increased mortality in patients with prolonged ED LOS observed in the study. Another potential explanation for worsening ICU outcomes is that the quality of critical care may have decreased. Even before the pandemic, Korea reported a mismatch between the supply of ICU beds and the increasing demand, along with a shortage of ICU staff [51–53]. In this context, allocating ICU beds to critically ill patients with COVID-19 could lead to a reduction in the available ICU capacity and potential overcrowding. A multicenter study of tertiary hospitals in Korea reported a decrease in the availability of ICU beds for non-COVID-19 patients [41]. A study of ICU registries from 15 countries found that ICU mortality was higher in systems that were already under-resourced before the pandemic and were unable to cope with the increased demand for critical care caused by the pandemic [14]. Although confounding factors such as demographics, scale of the COVID-19 pandemic, and public health measures could limit the findings, this suggests that differences in critical care resources, including ICU bed capacity and ICU nurse-to-patient ratios, may have a bearing on ICU outcomes during the pandemic. However, further studies are required to confirm these hypotheses.

To the best of our knowledge, this is the first nationwide study to investigate unplanned ICU admissions in Korea during the pandemic. Our findings have implications for critical care system planning and future pandemic management. Despite vast efforts to maintain the continuity of health services in response to the pandemic in Korea, we observed a decline in ED-to-ICU admissions and an increase in mortality, which implies reduced accessibility to critical care and deteriorated care quality. This decline in ED-to-ICU admissions may be partly related to the decreased demand for critical care during the pandemic. However, this decline is largely attributable to the failure of the critical care system’s capacity and resources. Furthermore, the response to the pandemic may have caused more unintended harm than the pandemic itself [54], particularly in environments with limited critical care resources [14]. Considering the widespread disruption of critical care and increased mortality in the early stages of the pandemic, as demonstrated in this study, our findings call for a reexamination of pandemic preparedness and the healthcare system’s response. These include expanding the ICU bed capacity in response to public health crises [15, 55], establishing regional coordination centers [56], and implementing protocols for determining ICU priority regardless of COVID-19 status [57]. Additionally, policies to enhance efficiency and reduce LOS in EDs are crucial, such as streamlining testing protocols [58], increasing staffing [59], and investing in telemedicine solutions [60].

This study had several limitations. First, a definitive causal relationship could not be established owing to the observational study design. Although the analysis was designed to strengthen certain key aspects of causality (temporal sequence, reversibility, strength of association, and coherence), residual confounding factors or other unmeasured aspects such as changes in ICU admission criteria not available in the data could mar the results. However, we implemented measures to reduce residual confounding factors and other unmeasured aspects by adjusting for seasonality in a setting where a randomized controlled trial is not feasible. Despite these limitations, our study provides important insights into the relationship between the pandemic and critical care and could inform future research efforts to definitively establish causality. Second, this study was not designed to reflect the surge in COVID-19 cases during the pandemic. In particular, the study did not include detailed data on ICU bed capacity, ICU occupancy rate, or staffing during the study period as these variables were not available in the NEDIS. Further research is needed to evaluate the changes in ICU admission and mortality in response to repeated surges during the COVID-19 pandemic. Third, we analyzed the NEDIS data from February 2018 to January 2021. However, the findings of our study may not be generalizable to later time periods, as the characteristics of the COVID-19 pandemic have changed significantly since then. For instance, the wild-type strain was the dominant SARS-CoV-2 variant in Korea until March 2021, but the Delta and Omicron variants have since emerged as the dominant strains globally [61]. These variants have been associated with an increase in ED presentations and ICU admissions among COVID-19 patients [62–64]. Therefore, future studies are needed to investigate the manner in which these variants have impacted ED-to-ICU admissions and patient outcomes.

## Conclusion

The COVID-19 pandemic exerted a significant impact on Korea’s critical care system. The number of patients admitted to the ICU through the ED decreased significantly, and the in-hospital mortality rate increased during that period. These challenges highlight the importance of monitoring ICU admissions and mortality rates during public health emergencies and the need for interventions to mitigate the impact of these events on patients with critical conditions.

### Electronic supplementary material

Below is the link to the electronic supplementary material.

Supplementary Material 1

## Data Availability

The datasets used and/or analyzed during the current study are available from the corresponding author on reasonable request.

## Abbreviations

CCI: Charlson Comorbidity Index
CI: Confidence interval
COVID-19: Coronavirus disease 2019
ED: Emergency department
EMS: Emergency medical service
ICD-10: International classification of diseases, tenth revision
ICU: Intensive care unit
IQR: Interquartile range
KTAS: Korean Triage and Acuity Scale
LOS: Length of stay
NEDIS: National Emergency Department Information System
NEWS: National Early Warning Score
NPI: Non-pharmaceutical intervention
RR: Relative risk
SARS-CoV-2: Severe acute respiratory syndrome coronavirus 2
US: United States
