# Supplementary material for: Impact of the early phase of the COVID-19 pandemic on emergency department-to-intensive care unit admissions in Korea: an interrupted time-series analysis
Source: BMC Emerg Med. 2024 Apr 1;24:51. doi: 10.1186/s12873-024-00968-1 (PMC10985913; doi:10.1186/s12873-024-00968-1)
Supplement: Supplementary file 1 — Supplementary Material 1 [file 12873_2024_968_MOESM1_ESM.docx]

**Additional File 1**

**Title**:

Impact of the early phase of the COVID-19 pandemic on emergency department-to-intensive care unit admissions in Korea: An interrupted time-series analysis

**Authors**:

Kyung-Shin Lee, Changwoo Han, Hye Sook Min, Jeehye Lee, Seok Hwa Youn, Younghwan Kim, Jae Young Moon, Young Seok Lee, Su Jin Kim, Ho Kyung Sung

**Corresponding author**:

Ho Kyung Sung, M.D.

National Emergency Medical Center, National Medical Center, Seoul, Korea

National Medical Center, 245 Eulgi-ro, Jung-gu, Seoul 04564, Korea

Tel: +82-2-6260-3180

Fax: +82-2-2260-7552

E-mail: hokyungsung@nmc.or.kr

**Supplementary Figure 1. Number of level 1 or 2 ED presentations and confirmed COVID-19 cases before and during the COVID-19 pandemic**


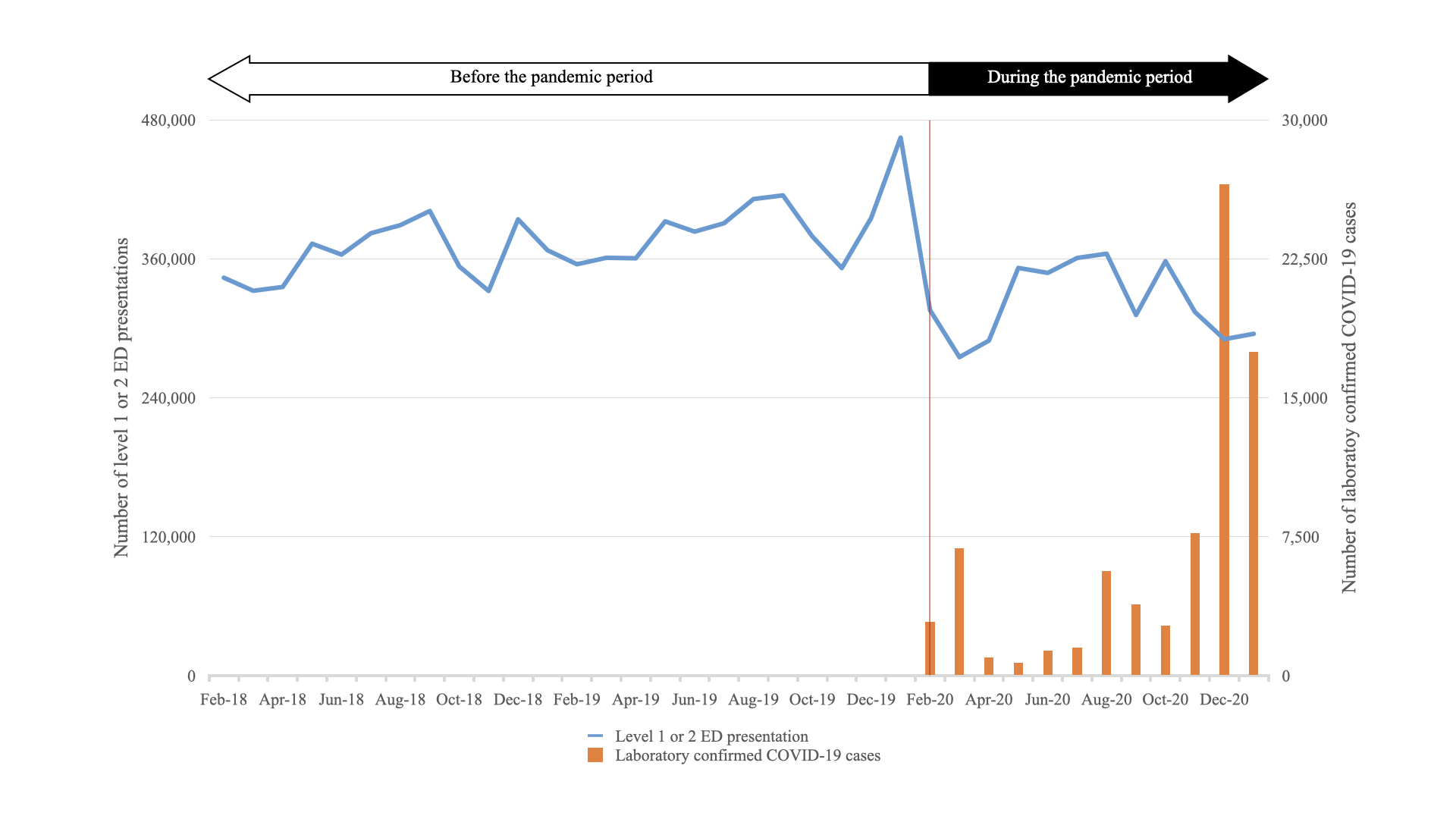


ED, emergency department; COVID-19, coronavirus disease **Supplementary Figure 2. Monthly median and percentage of prolonged ED LOS for the participants before and during the COVID-19 pandemic**

|  |  |
| --- | --- |

A. Median ED LOS (h)


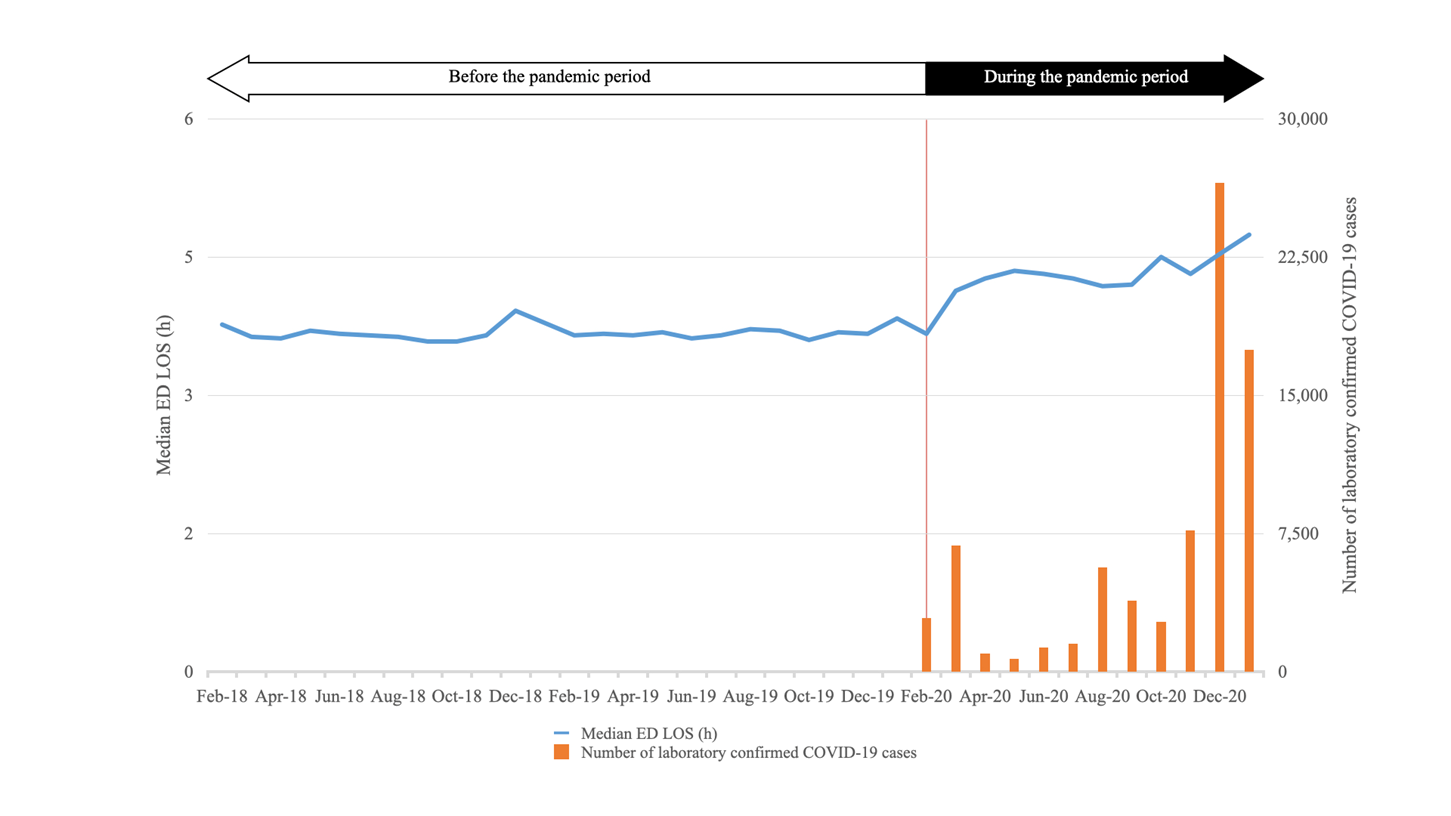


ED: emergency department, LOS: length of stay, COVID-19: coronavirus disease

B. Prolonged ED LOS (%)


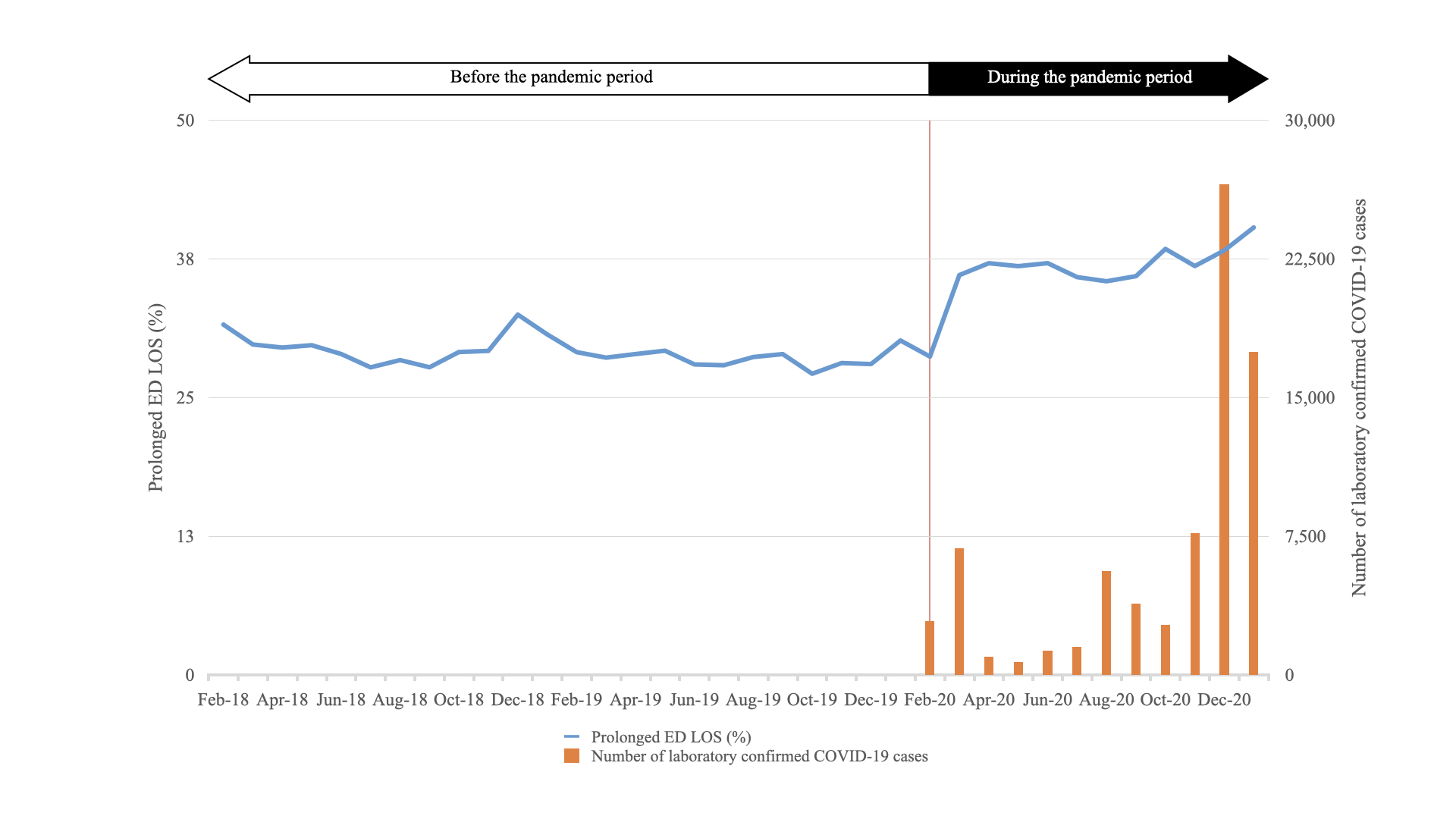


ED, emergency department; LOS, length of stay; COVID-19, coronavirus disease 2019

**Supplementary Table 1. Participation rate of EDs in the NEDIS**

|  | Overall level ED | | | Level 1 ED | | | Level 2 ED | | | Level 3 ED | | |
| --- | --- | --- | --- | --- | --- | --- | --- | --- | --- | --- | --- | --- |
|  | Total number of EDs in Korea | Number of EDs participating in the NEDIS | Participation rate (%) | Total number of EDs in Korea | Number of EDs participating in the NEDIS | Participation rate (%) | Total number of EDs in Korea | Number of EDs participating in the NEDIS | Participation rate (%) | Total number of EDs in Korea | Number of EDs participating in the NEDIS | Participation rate (%) |
| 2018 | 401 | 399 | 99.5 | 36 | 36 | 100 | 117 | 117 | 100 | 248 | 246 | 99.2 |
| 2019 | 402 | 401 | 99.8 | 38 | 38 | 100 | 124 | 124 | 100 | 240 | 239 | 96.5 |
| 2020 | 403 | 403 | 100 | 38 | 38 | 100 | 129 | 129 | 100 | 236 | 236 | 100 |
| 2021 | 398 | 398 | 100 | 38 | 38 | 100 | 127 | 127 | 100 | 233 | 233 | 100 |

ED, emergency department; NEDIS, National Emergency Department Information System

**Supplementary Table 2. Number of presentations at level 1 or 2 EDs, number of ED-to-ICU admissions, and ED-to-ICU admission rate before and during the COVID-19 pandemic**

|  | 2018 | | | 2019 | | | 2020 | | | 2021 | | |
| --- | --- | --- | --- | --- | --- | --- | --- | --- | --- | --- | --- | --- |
|  | Number of Level 1 or 2 ED presentation | Number of ICU admission | ICU admission rate (%) | Number of Level 1 or 2 ED presentation | Number of ICU admission | ICU admission rate (%) | Number of Level 1 or 2 ED presentation | Number of ICU admission | ICU admission rate (%) | Number of Level 1 or 2 ED presentation | Number of ICU admission | ICU admission rate (%) |
| January |  |  |  | 367,344 | 15,570 | 4.24 | 464,627 | 16,473 | 3.55 | 295,218 | 14,566 | 4.93 |
| February | 343,649 | 13,506 | 3.93 | 355,253 | 14,202 | 4.00 | 315,616 | 14,204 | 4.50 |  |  |  |
| March | 332,351 | 14,854 | 4.47 | 360,846 | 15,671 | 4.34 | 275,039 | 14,173 | 5.15 |  |  |  |
| April | 335,653 | 15,209 | 4.53 | 360,419 | 15,790 | 4.38 | 289,340 | 14,683 | 5.07 |  |  |  |
| May | 373,009 | 15,841 | 4.25 | 392,339 | 16,267 | 4.15 | 352,111 | 15,803 | 4.49 |  |  |  |
| June | 363,595 | 14,958 | 4.11 | 383,429 | 15,335 | 4.00 | 347,798 | 15,462 | 4.45 |  |  |  |
| July | 382,046 | 15,763 | 4.13 | 390,692 | 16,299 | 4.17 | 360,696 | 15,968 | 4.43 |  |  |  |
| August | 389,045 | 15,740 | 4.05 | 411,567 | 16,118 | 3.92 | 364,363 | 15,305 | 4.20 |  |  |  |
| September | 401,269 | 15,178 | 3.78 | 414,767 | 15,446 | 3.72 | 311,443 | 15,201 | 4.88 |  |  |  |
| October | 353,417 | 15,798 | 4.47 | 379,223 | 16,547 | 4.36 | 357,885 | 15,792 | 4.41 |  |  |  |
| November | 332,196 | 15,464 | 4.66 | 352,007 | 16,337 | 4.64 | 314,122 | 15,300 | 4.87 |  |  |  |
| December | 394,071 | 15,592 | 3.96 | 395,272 | 16,602 | 4.20 | 290,527 | 14776 | 5.09 |  |  |  |

Data are presented as number (%).

ED, emergency department; ICU, intensive care unit; COVID-19, coronavirus disease

**Supplementary Table 3. The ten most common primary diagnoses for ED-to-ICU admission before and during the COVID-19 pandemic**

|  | Before the COVID-19 pandemic | |  | During the COVID-19 pandemic | | |
| --- | --- | --- | --- | --- | --- | --- |
|  | Primary diagnosis | n (%) |  | Primary diagnosis | n (%) | *P* value^a^ |
| 1 | Acute myocardial infarction | 35,608 (9.5) | 1 | Acute myocardial infarction | 17,315 (9.6) | 0.573 |
| 2 | Intracranial injury | 27,563 (7.4) | 2 | Cerebral infarction | 14,143 (7.8) | 0.005 |
| 3 | Cerebral infarction | 27,064 (7.2) | 3 | Intracranial injury | 12,955 (7.2) | <0.001 |
| 4 | Intracerebral hemorrhage | 17,818 (4.8) | 4 | Intracerebral hemorrhage | 8,987 (5.0) | 0.001 |
| 5 | Pneumonia, organism unspecified | 17,207 (4.6) | 5 | Pneumonia, organism unspecified | 7,927 (4.4) | <0.001 |
| 6 | Subarachnoid hemorrhage | 11,108 (3.0) | 6 | Subarachnoid hemorrhage | 5,515 (3.0) | 0.1121 |
| 7 | Heart failure | 10,486 (2.8) | 7 | Heart failure | 4,914 (2.7) | 0.061 |
| 8 | Other sepsis | 8,099 (2.2) | 8 | Other sepsis | 4,375 (2.4) | <0.001 |
| 9 | Angina pectoris | 6,507 (1.7) | 9 | Acute renal failure^b^ | 3,035 (1.7)^b^ | <0.001 |
| 10 | Cardiac arrest | 6,006 (1.6) | 10 | Cardiac arrest | 2,619 (1.5) | <0.001 |

Data are presented as numbers (%).

ED, emergency department; ICU, intensive care unit; COVID-19, coronavirus disease

^a^*P* values for each diagnosis during the COVID-19 pandemic period were calculated relative to the same diagnosis before the COVID-19 pandemic period.

^b^The number and proportion of ED-to-ICU admissions for acute renal failure before the pandemic period were 5,339 and 1.4%, respectively.

**Supplementary Table 4. The top ten primary diagnoses with the highest number of deaths among ED-to-ICU admission cases before and during the COVID-19 pandemic**

|  | Before the COVID-19 pandemic | |  | During the COVID-19 pandemic | | *P* value^a^ |
| --- | --- | --- | --- | --- | --- | --- |
|  | Primary diagnosis | n (in-hospital mortality %) |  | Primary diagnosis | n (in-hospital mortality %) |  |
| 1 | Pneumonia, organism unspecified | 5,171 (30.1%) | 1 | Pneumonia, organism unspecified | 2,491 (31.4) | 0.028 |
| 2 | Intracranial injury | 3,829 (13.9) | 2 | Intracranial injury | 1,876 (14.5) | 0.112 |
| 3 | Intracerebral hemorrhage | 2,999 (16.8) | 3 | Intracerebral hemorrhage | 1,558 (17.3) | 0.299 |
| 4 | Cardiac arrest | 2,953 (48.9) | 4 | Other sepsis | 1,461 (33.4) | 0.662 |
| 5 | Other sepsis | 2,736 (33.8) | 5 | Cardiac arrest | 1,362 (52.0) | 0.015 |
| 6 | Acute myocardial infarction | 2,651 (7.4) | 6 | Acute myocardial infarction | 1,330 (7.7) | 0.334 |
| 7 | Cerebral infarction | 2,113 (7.8) | 7 | Cerebral infarction | 1,228 (8.7) | 0.002 |
| 8 | Subarachnoid hemorrhage | 1,933 (17.4) | 8 | Subarachnoid hemorrhage | 1,002 (18.2) | 0.222 |
| 9 | Shock, not elsewhere classified | 1,652 (39.5) | 9 | Shock, not elsewhere classified | 973 (39.5) | 0.991 |
| 10 | Heart failure | 1,443 (13.8) | 10 | Heart failure | 667 (13.6) | 0.752 |

Data are presented as numbers (%).

ED, emergency department; ICU, intensive care unit; COVID-19, coronavirus disease

^a^*P* values for each diagnosis during the COVID-19 pandemic were calculated relative to the same diagnosis before the COVID-19 pandemic.

**Supplementary Table 5. Multivariate logistic regression analysis for in-hospital mortality before and during the COVID-19 pandemic**

|  | Before the COVID-19 pandemic | | During the COVID-19 pandemic | |
| --- | --- | --- | --- | --- |
|  | aOR | 95% CI | aOR | 95% CI |
| Age group, y |  | |  | |
| 18–44 | Reference | | Reference | |
| 45–64 | 1.50 | 1.43–1.57 | 1.67 | 1.56–1.78 |
| 65–79 | 2.21 | 2.12–2.31 | 2.46 | 2.30–2.62 |
| 80 or older | 3.15 | 3.01–3.29 | 3.52 | 3.30–3.76 |
| Sex |  |  |  |  |
| Male | Reference | | Reference | |
| Female | 0.86 | 0.84–0.88 | 0.87 | 0.84–0.89 |
| Insurance type |  |  |  |  |
| NHI | Reference | | Reference | |
| Medical Aid | 1.06 | 1.03–1.09 | 1.03 | 0.99–1.08 |
| Uninsured or other | 1.15 | 1.08–1.23 | 1.28 | 1.16–1.42 |
| EMS presentation |  |  |  |  |
| No | Reference | | Reference | |
| Yes | 1.77 | 1.73–1.82 | 1.83 | 1.76–1.9 |
| Transferred-in |  |  |  |  |
| No | Reference | | Reference | |
| Yes | 1.17 | 1.14–1.19 | 1.11 | 1.08–1.15 |
| Injury-related presentation |  |  |  |  |
| No | Reference | | Reference | |
| Yes | 0.69 | 0.66–0.71 | 0.63 | 0.60–0.66 |
| KTAS score |  |  |  |  |
| 1 | Reference | | Reference | |
| 2 | 0.40 | 0.39–0.41 | 0.41 | 0.40–0.43 |
| 3 | 0.32 | 0.31–0.33 | 0.31 | 0.30–0.33 |
| 4 | 0.25 | 0.24–0.27 | 0.25 | 0.23–0.27 |
| 5 | 0.32 | 0.29–0.37 | 0.32 | 0.26–0.39 |
| Unidentified | 0.62 | 0.29–1.32 | 1.51 | 0.22–10.17 |
| NEWS |  |  |  |  |
| 0–4 | Reference | | Reference | |
| 5–6 | 2.00 | 1.94–2.06 | 2.01 | 1.93–2.10 |
| 7 or more | 2.78 | 2.71–2.86 | 2.73 | 2.63–2.83 |
| Unidentified | 2.39 | 2.33–2.46 | 2.43 | 2.34–2.52 |
| ED Length of stay |  |  |  |  |
| < 6h | Reference | | Reference | |
| ≥ 6h | 1.09 | 1.07–1.12 | 1.11 | 1.08–1.14 |
| CCI score |  |  |  |  |
| 0 | Reference | | Reference | |
| 1 | 0.83 | 0.81–0.85 | 0.81 | 0.78–0.84 |
| 2 | 0.81 | 0.78–0.83 | 0.73 | 0.70–0.77 |
| 3 or more | 1.02 | 0.98–1.05 | 0.94 | 0.90–0.99 |

aOR, adjusted odds ratio; CI, confidence interval; NHI, national health insurance; EMS, emergency medical service; KTAS, Korean Triage and Acuity Scale; NEWS, National Early Warning Score; ED, emergency department; LOS, length of stay; CCI, Charlson Comorbidity Index
